# Comparative research on the pipe-soil frictional resistances of circular and rectangular pipe sections during trenchless pipe jacking

**DOI:** 10.1371/journal.pone.0297537

**Published:** 2024-02-08

**Authors:** Jiwei Wen, Pengshuai Zhang, Tian Xiang

**Affiliations:** 1 Key Laboratory of Roads and Railway Engineering Safety Control (Shijiazhuang Tiedao University), Ministry of Education, Shijiazhuang, Hebei, China; 2 Key Laboratory of Metallogenic Prediction of Nonferrous Metals and Geological Environment Monitoring (Central South University), Ministry of Education, Changsha, Hunan, China; 3 State Key Laboratory of Geohazard Prevention and Geoenvironment Protection, Chengdu University of Technology, Chengdu, Sichuan, China; 4 Engineering Research Center of Geothermal Resources Development Technology and Equipment, Ministry of Education, Jilin University, Changchun, China; 5 China Construction Eighth Bureau Southwest Construction Engineering Co., Ltd., Chengdu, Sichuan, China; Jamia Millia Islamia, INDIA

## Abstract

Pipe jacking is a trenchless construction method to achieve forward tunneling and efficient construction of underground structure simultaneously without extensive surface excavation. In the process of pipe jacking construction, the jacking force provided by the hydraulic jacking equipment must overcome the frontal resistance of the cutter head and the frictional resistance between the pipe sections and formation at the same time. In particular, the pipe-soil frictional resistance increases with the increases of jacking distance, buried depth, pipe diameter and the complexity of jacking trajectory. Therefore, it is very important to correctly estimate jacking force in trenchless jacking engineering practice for the smooth implementation of pipe jacking, operation risk and comprehensive cost control. Firstly, the stress states of jacking circular and rectangular pipe sections in the soil are analyzed, and the key influencing factors of their pipe-soil frictional resistance are obtained respectively. Then, the pipe-soil frictional resistance of jacking the circular and rectangular pipe sections with the same external surface area in the dry sandy soil and coal granular layer are tested separately by using the self-developed multifunctional experimental apparatus during trenchless pipe jacking. The results show that the pipe-soil frictional resistances of jacking circular and rectangular pipe sections in the coal granular layer are always smaller than that in the sandy soil under the same experimental conditions, and the corresponding fitting calculation equation of pipe-soil frictional resistances are obtained respectively. Meanwhile, the modified calculation methods of the above pipe-soil frictional resistances are proposed respectively based on the relationship between the lateral pressure coefficient *K* and the buried depth of pipe section *H*. Moreover, the disturbed area of soil in the upper part of jacking circular pipe section presents an arc distribution, while the disturbed area of soil in the upper part of jacking rectangular pipe section presents a slightly concave distribution. Due to the different disturbance conditions of soil around the pipe section, the lateral pressure coefficient *K* should be corrected in the calculation equations of pipe-soil frictional resistance of jacking circular and rectangular pipe sections based on the discrete element numerical simulation analysis by EDEM software. Finally, the pipe-soil frictional resistances obtained by different methods in the sandy soil are compared and analyzed. The calculated values of the modified theoretical calculation method are very close to the experimental test values, while the other methods are smaller than the experimental test values, which makes the rationality of the modified theoretical calculation method of pipe-soil frictional resistance is verified, and some suggestions are also put forward for the value of some coefficients in the relevant empirical estimation equations. The above research achievements systematically compared the states of pipe-soil frictional resistances of jacking circular and rectangular pipe sections based on different research methods, especially for the correct evaluation of jacking force during trenchless pipe jacking, they could provide some valuable references and effective guidance for the subsequent research, engineering practice and further development of trenchless pipe jacking technology.

## Introduction

As one of the trenchless construction methods of underground structures, pipe jacking has been widely used in the high-efficiency construction of underground structures such as underground culverts, utility tunnel, underpass, tubular roof project, as well as underground coal mine roadway tunneling and relief well, etc. It has achieved remarkable social, economic and ecological benefits [1–5]. Compared with the traditional mining methods such as manual excavation and drilling-blasting method, pipe jacking has many significant advantages, including fast construction speed, strong formation adaptability, tunneling and supporting are carried out simultaneously, cut down material, safe working environment, etc. [1–9]. In particular, pipe jacking can make holes quickly and efficiently construct large-section underground rescue channels after accidents or disasters such as the collapse of coal mine roadway. It can play a very positive and vital role in avoiding or reducing the occurrence of serious underground accidents and disasters, as well as reducing casualties and property losses [3–7]. Moreover, it is different from the way that the power of the shield machine when it drives and moves forward in the formation comes from several hydraulic jacks arranged in the ring direction at the tail of the shield, in the practice of pipe jacking engineering, the main driving force for the complete set of pipe jacking machine and pipe sections to excavate and move forward in the formation comes from the jacking equipment set in the launching shaft at the end of the pipe sections. Once the pipe jacking machine and pipe sections enter the formation and start the jacking operation, the pipe jacking machine can only move forward but not back. Thus, the accurate estimation of jacking force is very important for the smooth implementation of trenchless pipe jacking operation [1–21].

In the process of pipe jacking, most of the jacking force provide by hydraulic jacking equipment must offset the frictional resistance between the outer wall of pipe section and the formation to achieve the whole pipe sections and pipe jacking machine tunneling and moving forward. The formation can be soil or rock, but the most cases are soil. Many pipe jacking engineering practices show that if the pipe-soil frictional resistance is too large, it may cause the engineering accidents such as jacking difficulty, pipe section cracking, the back support wall of launching shaft damage, etc. [1–13, 21] Thus, there are two key aspects to ensure the smooth implementation of trenchless pipe jacking operation as follow: (1) How to accurately estimate the jacking force for the construction design of pipe jacking operation? Currently, the main estimation methods of jacking force include theoretical analysis, experimental test, theoretical calculation equations, empirical estimation equations, and field monitoring, etc. However, each estimation method has its own applicable conditions and limitations, it is difficult to provide a general guidance for pipe jacking engineering practice [1–5, 10–19]. (2) How to effectively reduce the pipe-soil frictional resistance during pipe jacking process? At present, the common practice is to inject thixotropic mud into the gap between the outer wall of pipe sections and the formation, so that the slurry jacket is formed around the outer wall of pipe sections to achieve the effect of lubrication and friction reduction [1–5, 9–12, 16].

Ma proposed a new pipe-soil contact model for rectangular pipe-jacking for friction force prediction under the lubricant applied condition, and a numerical simulation was conducted using the finite element method to verify the proposed model [10]. Ye provided a new approach to predict the friction resistance of slurry pipe jacking, the influence of buried depth, overcut, and pipe diameter on the friction resistance and lubrication efficiency were analyzed [16]. Kong discussed the frictional resistance of rectangular pipe jacking under the pipe-slurry-soil contact state by using theoretical calculation, field monitoring and FLAC ^3D^ numerical simulation, the research results are in good agreement with the actual situation [21]. Herein, the slurry for friction reduction has not been considered for the time being. Moreover, the differences between the circular and the rectangular are also analyzed. This study aims to investigate the pipe-soil frictional resistance of jacking circular and rectangular pipe sections under the various working conditions by using theoretical analysis, experimental test, discrete element numerical simulation analysis and other methods. The multifunctional experimental apparatus for pipe-soil frictional resistance test in the process of trenchless pipe jacking was independently developed, which can provide the necessary research conditions for physical model experiment. Moreover, the results of the experimental test, the frequently used theoretical calculation equations and empirical estimation equations, and the discrete element numerical simulation analysis by EDEM software are compared and discussed. The above research contents mentioned in this paper are all the authors’ original innovation research based on the previous research. These research achievements could provide some valuable guidance for the further pipe jacking engineering practice, scientific research, and technological innovation and progress.

## Technical principle of trenchless pipe jacking

**Fig 1:**
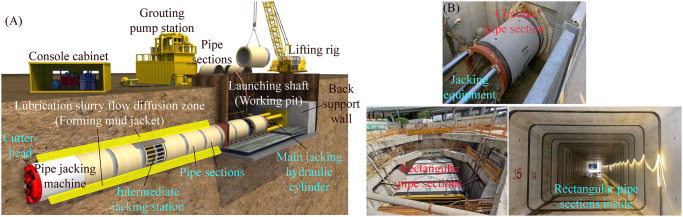
Schematic diagram of trenchless pipe jacking principle and its actual operation scenario. (A) Schematic diagram of trenchless pipe jacking principle [1]. (B) Operation scenario of jacking circular pipe section. (C) Operation scenario of jacking rectangular pipe section.

As shown in Fig 1, trenchless pipe jacking is to use the jacking force provided by the main jacking hydraulic cylinder set in the launching shaft (working pit) and the several intermediate jacking stations are arranged between the adjacent pipe sections, to drive the pipe jacking machine tunneling from the launching shaft continuously through the formation in accordance with the designed trajectory until it reaches the arriving shaft (receiving pit). It can synchronously complete the trenchless laying of pipe sections one by one immediately following the back of the pipe jacking machine. The whole trenchless pipe jacking construction process of underground structures does not require extensive excavation of the surface [1–7]. Currently, the cross-section shape of pipe section used for trenchless pipe jacking is mainly circular and rectangular. Moreover, under the premise of the same effective space, the rectangular pipe section can save more than one third of the underground space compared with the circular pipe section, and the former one can greatly reduce the tunnel depth and increase the thickness of the overlying soil layer. Thus, the rectangular pipe jacking construction has obvious advantages in saving construction investment cost and reducing construction safety risk. From the comprehensive consideration of usable function and economic performance, the underground structures with rectangular cross-section shape composed of several rectangular pipe sections have more extensive application prospects in the practice [3–5, 18, 20].

**Fig 2:**
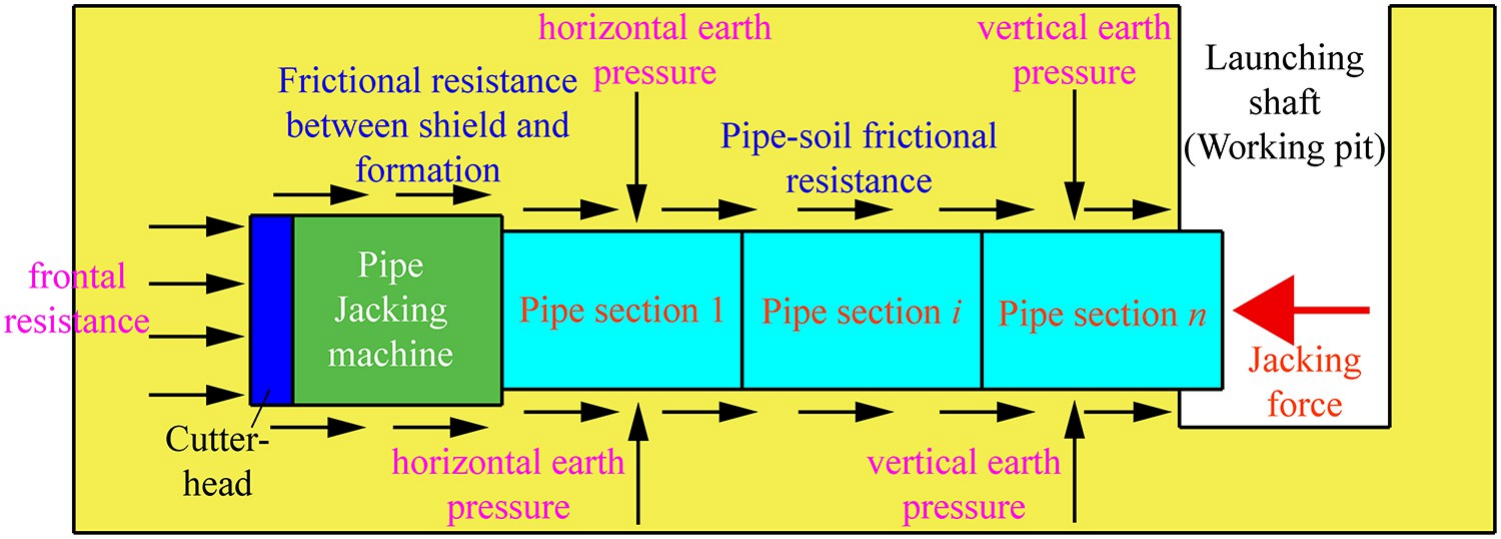
Schematic diagram of main force conditions of jacking mechanical tools in the process of trenchless pipe jacking.

As shown in Fig 2, in the process of pipe jacking, the jacking force provided by the jacking equipment is the power source for pushing all pipe sections and pipe jacking machine forward tunneling and moving. The jacking force not only needs to overcome the head-on (penetration) resistance of the cutter head of the pipe jacking machine, but also must overcome the frictional resistance between the outer wall of the pipe sections (including pipe jacking machine) and the formation, especially the pipe-soil frictional resistance often consumes most of the jacking force. It is more obvious with the longer jacking distance, larger pipe diameter, larger soil cover depth and more complex jacking trajectory. Thus, in the practice of trenchless pipe jacking engineering, pipe-soil frictional resistance is the key factor to be considered when determining the jacking force of equipment. The reasonable estimation of jacking force directly determines the success or failure of trenchless pipe jacking operation, it also has great influence on the aspects of safety, resource allocation and comprehensive cost control during operation [1–5, 10–19].

### Theoretical force analysis of pipe section during trenchless jacking circular and rectangular pipe sections in the soil

#### Force analysis of jacking circular pipe section in the soil

**Fig 3:**
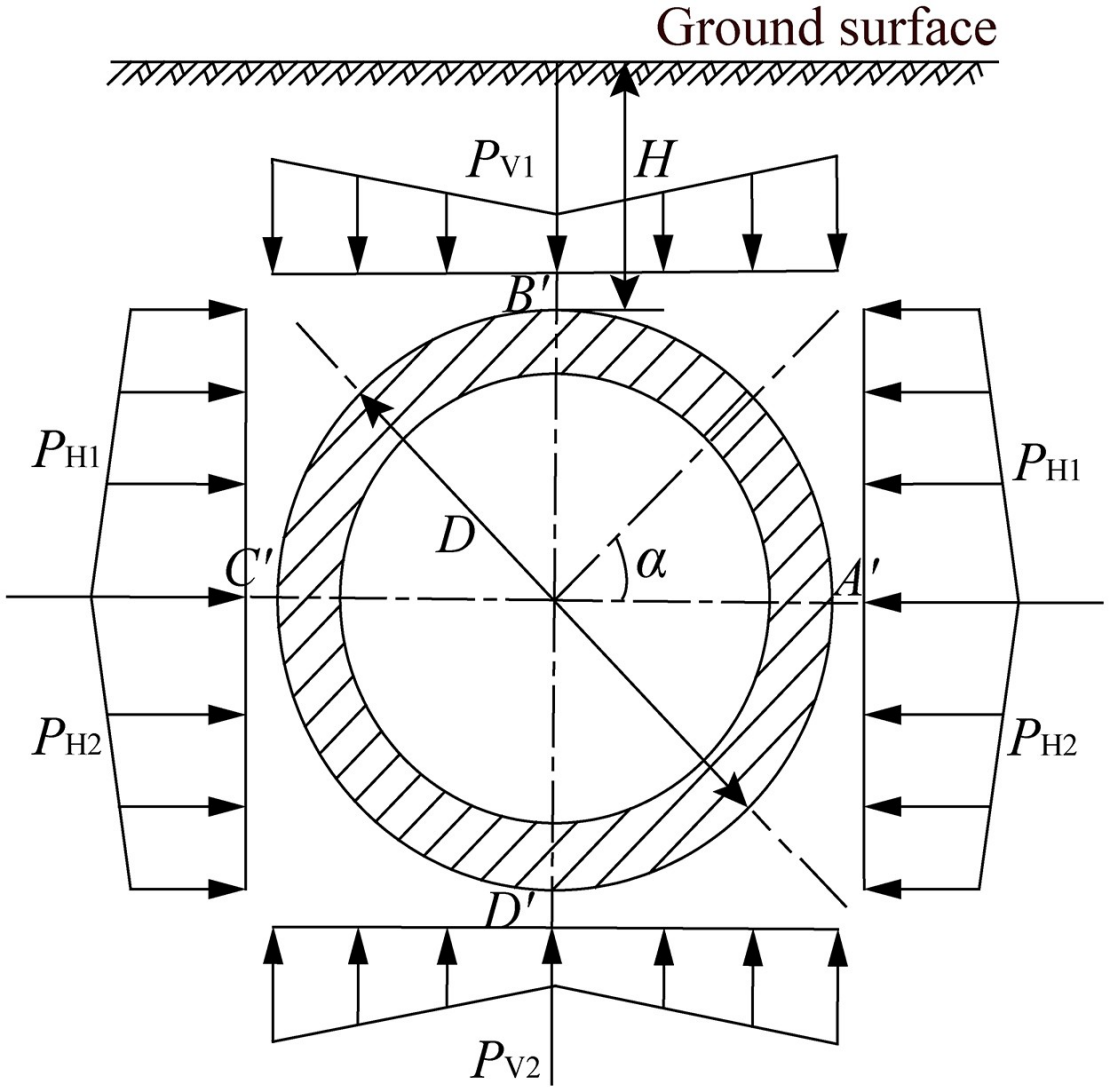
Schematic diagram of force state of jacking circular pipe section in the soil [17].

The force state of jacking circular pipe section in the soil is shown in Fig 3. The outer diameter of the circular pipe section is *D*, and the buried depth from the middle of the upper surface of pipe section to the surface is *H*. If the weight of the pipe section is not considered, then the force state of the circular pipe section has the characteristics of up and down and left and right symmetry. In the upper and lower parts of the pipe section, the actual buried depth of the pipe section gradually increases from the top to the side of the pipe section, so the soil pressure at the top of the pipe section is a gradual function of the calculated point position. In the same way, the lateral pressure is also a gradual function of the calculated point position. The pipe-soil frictional resistance of jacking circular pipe section in the soil can be calculated as the following theoretical calculation equation [17]:

$$F_{c}=fL[\frac{\pi }{2}\gamma D(1+K)\left(H+\frac{D}{2}\right)-\frac{1}{3}\gamma D^{2}(2+K)]+f\omega L$$ (1)

Where, *F*_c_ is the pipe-soil frictional resistance of jacking circular pipe section in the soil (N), *f* is the frictional coefficient between the outer wall of pipe section and the surrounding soil, *L* is the length of contact between the outer wall of pipe section and soil (m), *D* is the outside diameter of the circular pipe section (m), *K* is the lateral soil pressure coefficient, *H* is the thickness of covering soil above the top of pipe section (m), *γ* is the volumetric weight of soil (kN/m^3^), *ω* is the linear density of pipe section (N/m).

#### Force analysis of jacking rectangular pipe section in the soil

**Fig 4:**
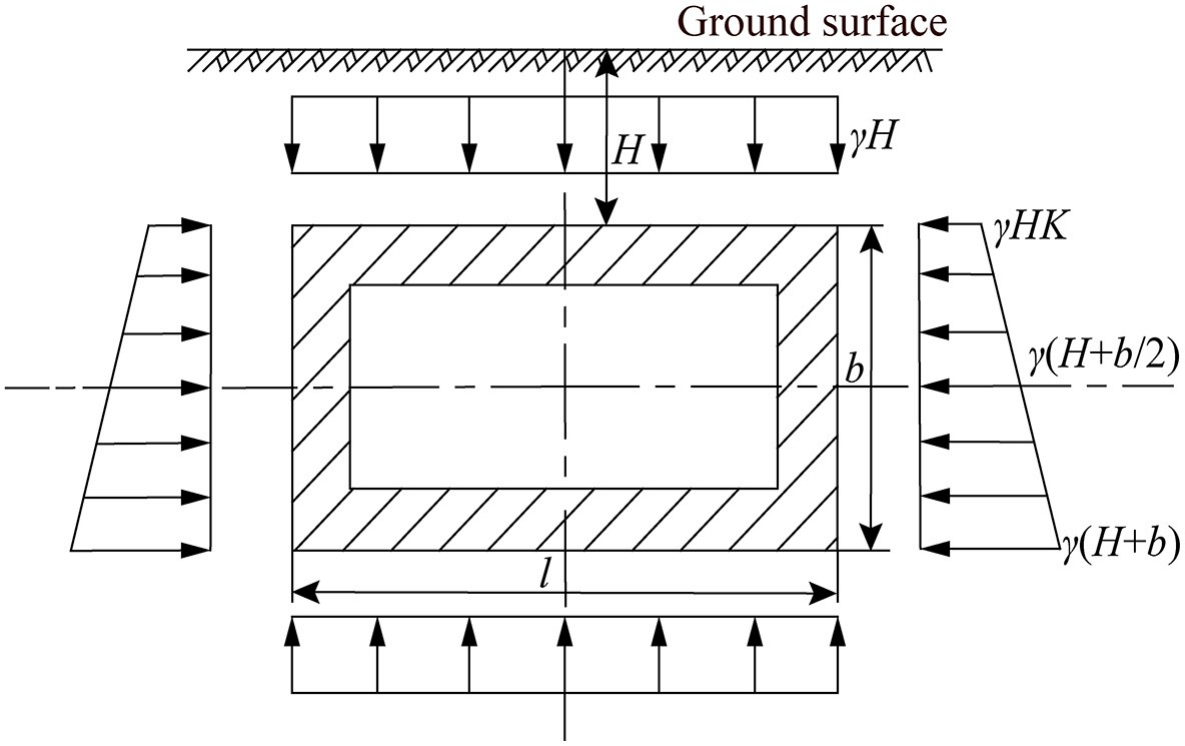
Schematic diagram of force state of jacking rectangular pipe section in the soil [18].

The force state of jacking rectangular pipe section in the soil is shown in Fig 4, it is symmetrical on the upper and lower sides and the left and right sides respectively. The length and width of the rectangular pipe section are *l* and *b*, respectively. The soil pressure in the upper and lower part of the pipe section remains constant, and the lateral soil pressure from the top to the side of pipe section is trapezoidal [18].

The pipe-soil frictional resistance of jacking rectangular pipe section in the soil can be calculated as the following theoretical calculation equation [18]:

$$F_{r}=fL\left[2\gamma Hl+2K\gamma Hb+\gamma b^{2}K\right]+f\omega L$$ (2)

**Fig 5:**
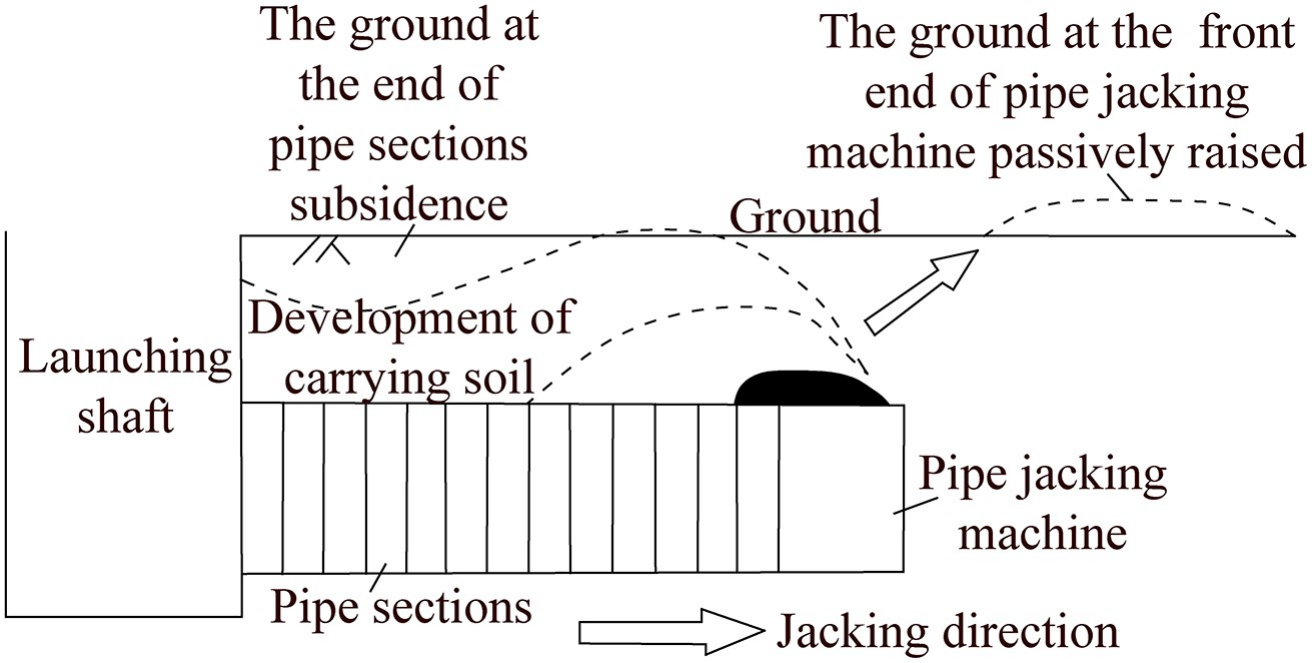
Schematic diagram of carrying soil effect during jacking pipe section in the soil [20].

From the above force states and theoretical calculation equations, it is not difficult to find that the pipe-soil frictional resistances of the circular and rectangular pipe sections are all determined by the following crucial factors: the pipe section specification, the frictional coefficient between the outer wall of pipe section and the surrounding soil, the volumetric weight of soil, the buried depth of pipe section, the lateral soil pressure coefficient, the length of contact between the outer wall of pipe section and soil. Moreover, due to the difference in shape between circular and rectangular pipe sections, the conditions of soil pressure distribution on their outer walls are different, so the corresponding theoretical calculation equations of pipe-soil frictional resistance are also different. It is necessary to note that the soil in the above two theoretical calculation equation is assumed to be an ideal rigid body with no deformation characteristics. However, the actual process of pipe jacking is dynamic, the pipe-soil frictional resistance will drive the soil around the pipe section to move along the jacking direction, and the soil around the outer wall of pipe section will cause disturbance and shear deformation, which is also called the carrying soil effect as shown in Fig 5 [20].

In fact, the carrying soil effect will cause changes in the soil pressure around the pipe section, which is reflected in the above two theoretical calculation equations, namely the value of lateral soil pressure coefficient *K*. To facilitate calculation, it is generally believed that the lateral soil pressure coefficients *K* in the above two equations are taken as the active earth pressure, i.e., *K*_a_ = tan^2^(45°-*φ*/2). However, because the jacking pipe section is a dynamic process, the various shape of pipe sections will cause different disturbance conditions of the surrounding soil, and this influence will also vary in different formation and under different buried depth *H*. Thus, the lateral soil pressure coefficient *K* is uncertain, and it cannot be simply considered that its value is the active earth pressure coefficient under any circumstances. To calculate the pipe-soil frictional resistance when jacking the circular and rectangular pipe sections in soil more accurately, the corresponding test experiments are carried out under the different buried depth of pipe sections. The relationship between the buried depth of pipe section *H*, the pipe-soil frictional resistance *F* and the lateral soil pressure coefficient *K* during pipe jacking in different soil is investigated, so as to obtain a more accurate calculation method of pipe-soil frictional resistance. It can provide the scientific and valuable reference for jacking force calculation in the pipe jacking practice.

### Experimental test of the pipe-soil frictional resistance during trenchless pipe jacking

#### Multifunctional experimental apparatus for the pipe-soil frictional resistance testing

**Fig 6:**
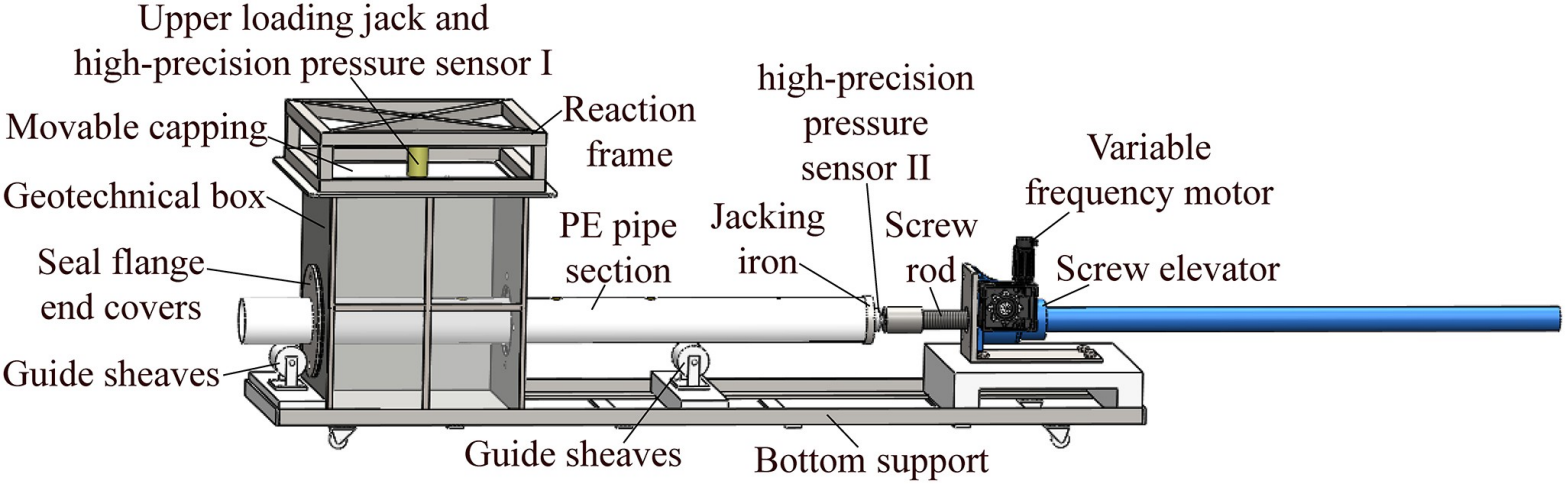
3D model diagram of multifunctional apparatus for the pipe-soil frictional resistance testing.

**Fig 7:**
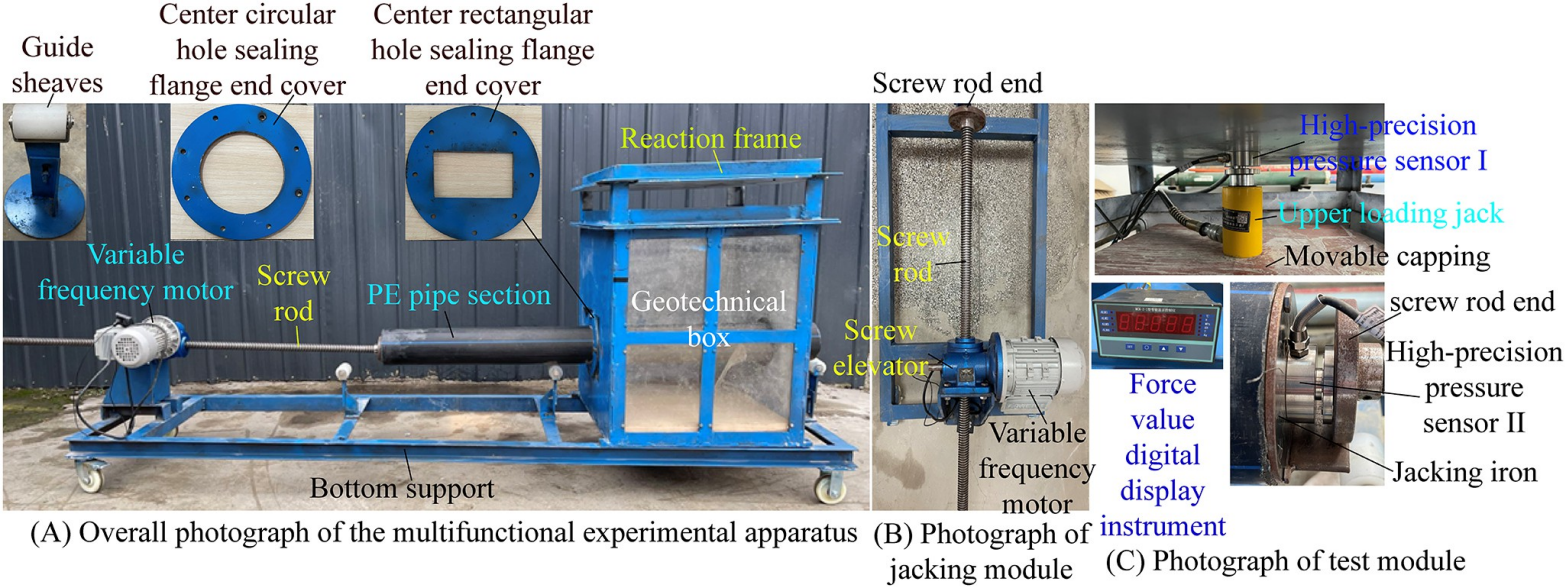
Physical photograph of multifunctional apparatus for the pipe-soil frictional resistance testing.

The 3D model and physical photograph of experimental apparatus for pipe-soil frictional resistance testing are shown in Figs 6 and 7, respectively. It is mainly composed of geotechnical box module, pipe section, jacking module and test module. Among them, the geotechnical box module is composed of geotechnical box, sealing flange end covers, guide sheaves, bottom support, reaction frame, movable capping and upper loading jack. The geotechnical box is used to contain the simulated formation required for experimental testing, and its specification is 0.6 m (length)×0.6 m (width)×0.7m (height). A movable capping is placed on the simulated formation surface, and a movable hydraulic jack connected to the upper reaction frame is set in the middle of the capping. The jack exerts normal force on the capping, and then the concentrated normal load is changed into uniform load from the capping to act on the simulated formation surface. By regulating the overlying load of pipe section, it plays the role of indirectly adjusting the buried depth of the pipe section, i.e., the overlying formation pressure. The high-precision pressure sensor I is arranged between the jack and the reaction frame, and the pressure value fed back in real time by the force value digital display instrument is observed, and then the load value applied by the jack is dynamically and accurately regulated. The guide sheaves are used to support the stable migration of pipe section and prevent it from deformation due to its own weight. Moreover, the Polyethylene (PE) pipes with circular and rectangular section shapes are selected as the experimental pipe sections. To better investigate the pipe-soil frictional resistances of the two kinds of pipe sections, they should have the same surface area, i.e., the same pipe-soil contact surface area, here is 0.7 m^2^. Among them, the circular pipe section specifications are 140 mm (outer diameter) ×1600 mm (pipe length) ×13 mm (wall thickness), and the rectangular pipe section specifications are 140 mm (length) ×80 mm (width) ×1600 mm (pipe length) ×13 mm (wall thickness). The jacking module is used to provide the jacking force when the pipe section is jacking forward in the simulated formation, it mainly includes the screw elevator, the variable frequency motor, and the jacking iron. The model of the screw elevator is SWL2.5, it is bolted to the bottom support, its transmission ratio is 6:1, and the total length of the screw is 1.5 m. The model of the variable frequency motor is Y2 71-4P-0.55kW-B5, it is attached to the side of the screw elevator to deliver power. Its speed regulation range is between 430~1400 r/min, that is, the movement speed of the screw is 0.43~1.4 m/min, and the uniform speed of the pipe section can be maintained during the experimental test. The screw shaft is aligned with the center of the hole at the side end of the geotechnical box, and the jacking iron is a circular iron sheet with a diameter slightly larger than the pipe section, which is fixed by the several bolts on the end of the pipe section bearing the jacking force, and a high-precision pressure sensor II is arranged between the end of the screw and the jacking iron.

#### Methods of the experimental test

The main variable of the experimental test is the buried depth *H* of the pipe section, it is divided into five working conditions of 0.3 m, 0.45 m, 0.6 m, 0.75 m, and 0.9 m. The pipe-soil frictional resistances of jacking circular and rectangular pipe sections in the dry sandy soil and the dry coal granular layer are tested respectively. Among them, when the buried depth *H* = 0.3 m is the true thickness of the filled soil, while when the buried depth *H* is greater than 0.3 m, due to the limitation of the specifications of the geotechnical box, the purpose of changing the buried depth *H* (the overlying soil pressure) should be adopted by adjusting the normal load jointly imposed by the movable hydraulic jack and the capping. The conversion relationship between the buried depth *H* and the normal load of the jack can be calculated by the following equation:

$$H=\frac{\left(M+\frac{N}{g}\right)g}{S\cdot \gamma }$$ (3)

Where, *H* is the buried depth of the pipe section (m); *M* is the total masses of soil, capping and jack above the pipe section (kg); *N* is the normal load applied by the jack (N); *g* is the gravitational acceleration (9.8 m/s^2^); *S* is the cross-sectional area of the chamber in the geotechnical box (m^2^); *γ* is the volumetric weight of soil (kN/m^3^).

**Fig 8:**
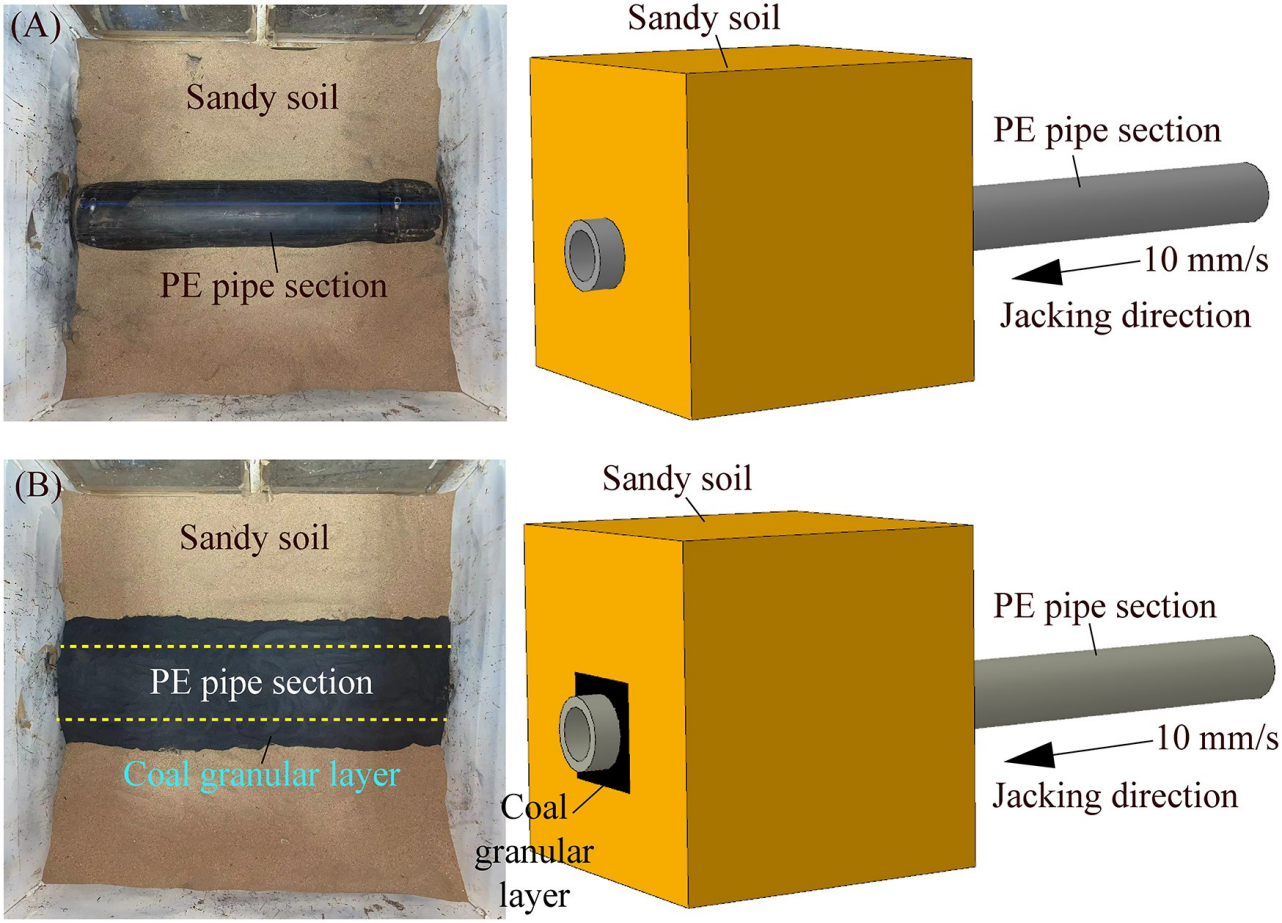
Experimental test conditions of jacking pipe section in the various soil layers. (A) Experimental condition of jacking pipe section in sandy soil. (B) Experimental condition of jacking pipe section in coal granular layer.

The experimental test conditions of jacking pipe section in sandy soil and coal granular layer are shown in Fig 8. The pipe section is through the geotechnical box before the experimental test, and the pipe end is exposed 5 cm along the jacking direction of the geotechnical box. The jacking speed is set at 10 mm/s and maintained at a constant speed. The test ended after the pipe section is jacked forward by 0.9 m. According to the screening test, the particle size of the coal granular layer used in the experimental test ranges from 0.075mm to 0.25mm, and the particle size composition of the sandy soil used in the experimental test is shown in Table 1. To ensure the smooth implementation of pipe-soil frictional resistance testing of jacking in coal granular layer, the amount of coal particles is reduced as much as possible, the 50 kg of coal particles are mixed in the middle of sandy soil and evenly coated around the outer wall of pipe section. Moreover, per the mass and volume of the soil filled in the geotechnical box, the volumetric weights of the sandy soil and the coal-sandy soil layer can be calculated as 18610 N/m^3^ and 18105 N/m^3^ respectively.

**Table 1:** Particle size composition of sandy soil for experimental testing.

Particle size distribution / mm	Mass percent / %
0.5~2	12.5
0.25~0.5	53.8
0.075~0.25	25.9
< 0.075	7.8

## Results and analysis of the experimental test

### Experimental test results of the pipe-soil frictional resistance

**Fig 9:**
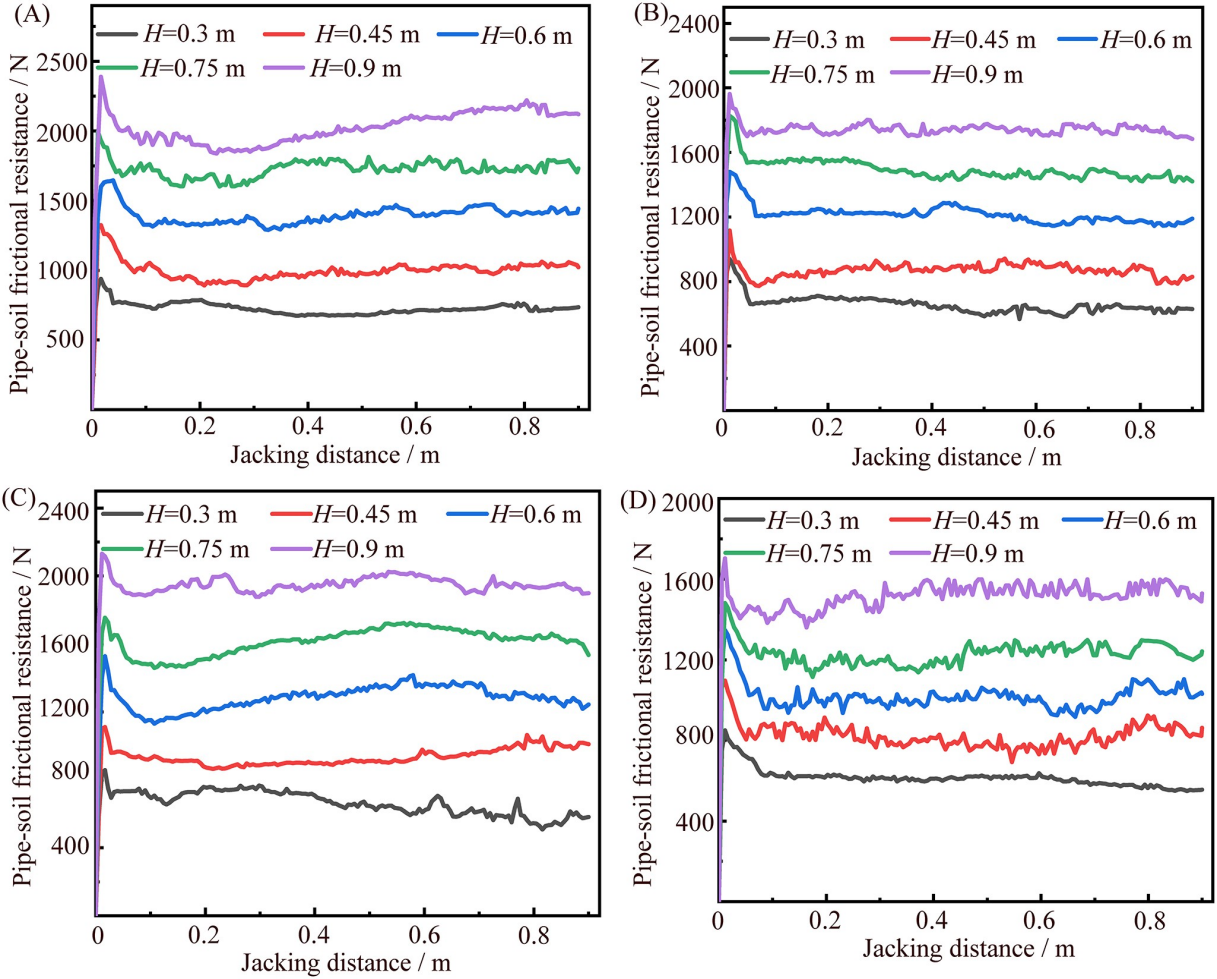
Relationship curves between pipe-soil frictional resistance and jacking distance of jacking circular and rectangular pipe sections under the different experimental conditions. (A) Relationship curves between pipe-soil frictional resistance and jacking distance of jacking circular pipe section in sandy soil at different buried depth. (B) Relationship curves between pipe-soil frictional resistance and jacking distance of jacking circular pipe section in coal granular layer at different buried depth. (C) Relationship curves between pipe-soil frictional resistance and jacking distance of jacking rectangular pipe section in sandy soil at different buried depth. (D) Relationship curves between pipe-soil frictional resistance and jacking distance of jacking rectangular pipe section in coal granular layer at different buried depth.

The relationship curves between pipe-soil frictional resistance and jacking distance during jacking circular and rectangular pipe sections in sandy soil and coal granular layer at the various buried depth *H* is shown in Fig 9. We can find that the relationship curves between pipe-soil frictional resistance and jacking distance under the different experimental conditions all rapidly reach the peak value of pipe-soil frictional resistance and then decrease and gradually become stable. This is because in the initial stage of jacking pipe sections in the simulated formation, it will experience a short acceleration process from static to forward migration, and then it will keep a constant speed, and the pipe-soil frictional resistance will gradually change from static friction to dynamic friction. Among them, the peak value of pipe-soil frictional resistance is the maximum static frictional resistance, and the stabilizing stage can be regarded as the sliding friction resistance.

The experimental test values obtained in Fig 9 under the different experimental conditions are respectively averaged and recorded as the corresponding pipe-soil frictional resistance, the results are shown in Table 2. Under the experimental condition of full contact between pipe sections and soil, the pipe-soil frictional resistances of jacking the various pipe sections in coal granular layer are all smaller than that of jacking them in sandy soil. Moreover, the difference of pipe-soil frictional resistances of jacking pipe sections in the above two kinds of simulated formation also increases with the increase of buried depth. Herein, the maximum difference of jacking the circular pipe section in different simulated formation is up to 274 N, the maximum difference of jacking the rectangular pipe section is up to 428 N. It is indicated that the pipe-soil frictional resistances during pipe jacking in the various formations are very different. Thus, the relevant technical parameters of pipe jacking should be adjusted based on the corresponding formation. For example, if the construction design is carried out based on the technical parameters of pipe jacking in sandy soil by using engineering analogy method, the jacking force provided by the jacking equipment can be appropriately reduced due to the reduction of pipe-soil frictional resistance in coal granular layer, so as to reduce the comprehensive cost of pipe jacking operation to a certain extent.

**Table 2:** Average pipe-soil frictional resistance of jacking circular and rectangular pipe sections under the different experimental conditions.

Pipe section type	Buried depth *H* / m	Simulated formation	Average pipe-soil frictional resistance *F*_a_ / N	Difference / N
Circular pipe section	0.3	Sandy soil	631	50
		Coal granular layer	581	
	0.45	Sandy soil	930	65
		Coal granular layer	865	
	0.6	Sandy soil	1304	95
		Coal granular layer	1209	
	0.75	Sandy soil	1670	185
		Coal granular layer	1485	
	0.9	Sandy soil	2006	274
		Coal granular layer	1732	
Rectangular pipe section	0.3	Sandy soil	625	126
		Coal granular layer	499	
	0.45	Sandy soil	927	177
		Coal granular layer	750	
	0.6	Sandy soil	1275	263
		Coal granular layer	1012	
	0.75	Sandy soil	1603	354
		Coal granular layer	1249	
	0.9	Sandy soil	1936	428
		Coal granular layer	1508	

**Fig 10:**
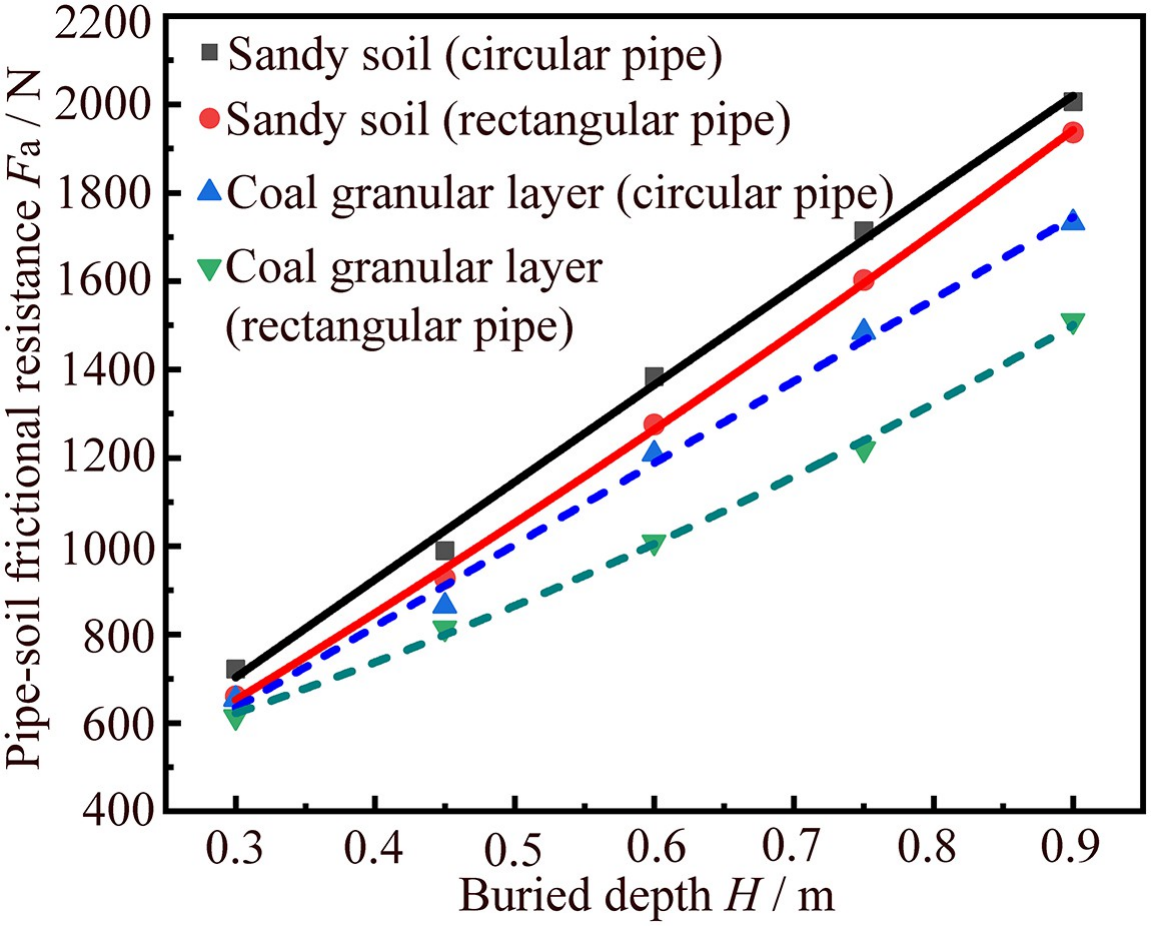
Relationship fitting curves between the average pipe-soil frictional resistance and pipe section buried depth of jacking circular and rectangular pipe sections in different soil layers.

The relationships between the average pipe-soil frictional resistances *F*_a_ and the buried depths *H* of jacking circular and rectangular pipe sections in sandy soil and coal granular layer in Table 2 are drawn into a scatter plot as shown in Fig 10, and the four corresponding fitting relationship can be obtained as the following equations:

(1) The pipe-soil frictional resistance of jacking the circular pipe section in sandy soil *F*_cs_:

$$F_{cs}=209.52H^{2}+2075.24H-21.8$$ (4)

(2) The pipe-soil frictional resistance of jacking the rectangular pipe section in sandy soil *F*_rs_:

$$F_{rs}=133.33H^{2}+2038.67H-4$$ (5)

(3) The pipe-soil frictional resistance of jacking the circular pipe section in coal granular layer *F*_cc_:

$$F_{cc}=450.79H^{2}+2488.95H-136.4$$ (6)

(4) The pipe-soil frictional resistance of jacking the rectangular pipe section in coal granular layer *F*_rc_:

$$F_{rc}=28.57H^{2}+1712.29H-12.2$$ (7)

From Fig 10 we can find that the goodness of fit of the above four fitting relationship equations are 0.999, 0.999, 0.998 and 0.999, respectively. Thus, it shows that the fitting results of these four equations are better. Moreover, the values of the pipe-soil frictional resistances *F*_cs_, *F*_rs_, *F*_cc_ and *F*_rc_ all tend to increase with the increase of buried depth *H*. The above four fitting relationship equations can be applied to actual pipe jacking engineering to preliminary estimate the pipe-soil frictional resistances of jacking the circular and rectangular pipe sections in sandy soil and coal granular layer respectively.

### Experimental test of the PE slab-soil frictional coefficient

**Fig 11:**
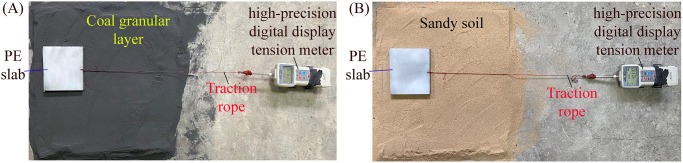
Experimental test conditions of PE slab-soil frictional coefficient. (A) Experimental test of the frictional coefficient between PE slab and coal granular layer. (B) Experimental test of the frictional coefficient between PE slab and sandy soil.

To better reflect the contact characteristics between the outer wall of the pipe section and formation, and obtain the frictional coefficient between the pipe sections and formation in the theoretical calculation equation, the experimental test of slab-soil frictional coefficient on the sandy soil and coal granular layer is carried out as shown in Fig 11. To facilitate experimental operation, a PE slab with a size of 140 mm×140 mm×15 mm and a mass of 0.33 kg is selected, and a rope hole is provided in the middle of its end, and then the traction rope is connected with a high-precision digital display tension meter with a roller at the bottom, so that the tension meter can move forward at a uniform speed on the flat ground. The PE slab is driven to slide the same distance at uniform speed on the flat sandy soil and coal granular layer on the upper surface respectively. Each group of experiments is repeated at least 3 times. The average tension value is recorded as the frictional resistance of PE slab during the sliding process of the sandy soil and coal granular layer surface. The experimental test is divided into 4 groups, the masses applied to the soil surface are 0.33 kg, 0.66 kg, 0.99 kg and 1.32 kg. It is adjusted by superimposing PE slabs with the same specification.

The experimental test results of PE slab-soil frictional coefficient are listed in Table 3. It can be seen that the frictional characteristics of PE slab in contact with sandy soil and coal granular layer are different. Under different loading conditions, the frictional coefficient between PE slab and sandy soil ranges from 0.45 to 0.49, and the average frictional coefficient is 0.474. Furthermore, the frictional coefficient between PE slab and coal granular layer ranges from 0.38 to 0.41, and the average frictional coefficient is 0.397. Thus, the frictional coefficient between PE slab and sandy soil is greater than the frictional coefficient between PE slab and coal granular layer.

**Table 3:** The experimental test results of PE slab-soil frictional coefficient.

Experimental number	Loading mass / kg	Frictional resistance / N		Frictional coefficient	
		Sandy soil	Coal granular layer	Sandy soil	Coal granular layer
1	0.33	1.58	1.36	0.48	0.41
2	0.66	2.98	2.58	0.45	0.39
3	0.99	4.81	4.02	0.49	0.41
4	1.32	6.33	5.01	0.48	0.38
Average value		3.925	3.243	0.474	0.397

### Modifications of the lateral pressure coefficient *K* and the theoretical calculation equations of pipe-soil frictional resistance

The experimental test results of the average pipe-soil friction resistance *F*_a_ obtained by jacking circular and rectangular pipe sections in sandy soil and coal granular layer are put into Eqs (1) and (2) respectively, Thus, the corresponding lateral pressure coefficient *K* under different buried depths *H* can be inversely calculated based on the Eqs (1) and (2). The detailed parameters used for theoretical calculation equations are shown in Table 4. Among them, *γ*_s_ is the volumetric weights of the sandy soil, *γ*_cs_ is the volumetric weights of the coal-sandy soil layer, *f*_s_ is the frictional coefficient between the PE pipe section and the sandy soil, *f*_c_ is the frictional coefficient between the PE pipe section and the coal granular layer, and the meanings of other parameters are consistent with those described in Eqs (1) and (2).

**Table 4:** The detailed parameters used for theoretical calculation equations of pipe-soil frictional resistance.

*γ*_*s*_ / N·m^-3^	*γ*_*cs*_ / N·m^-3^	*D* / m	*L* / m	*ω* / N·m^-1^	*f*_*s*_	*f*_c_	*l* / m	*b* / m
18610	18105	0.14	0.6	41.8	0.474	0.397	0.14	0.08

**Fig 12:**
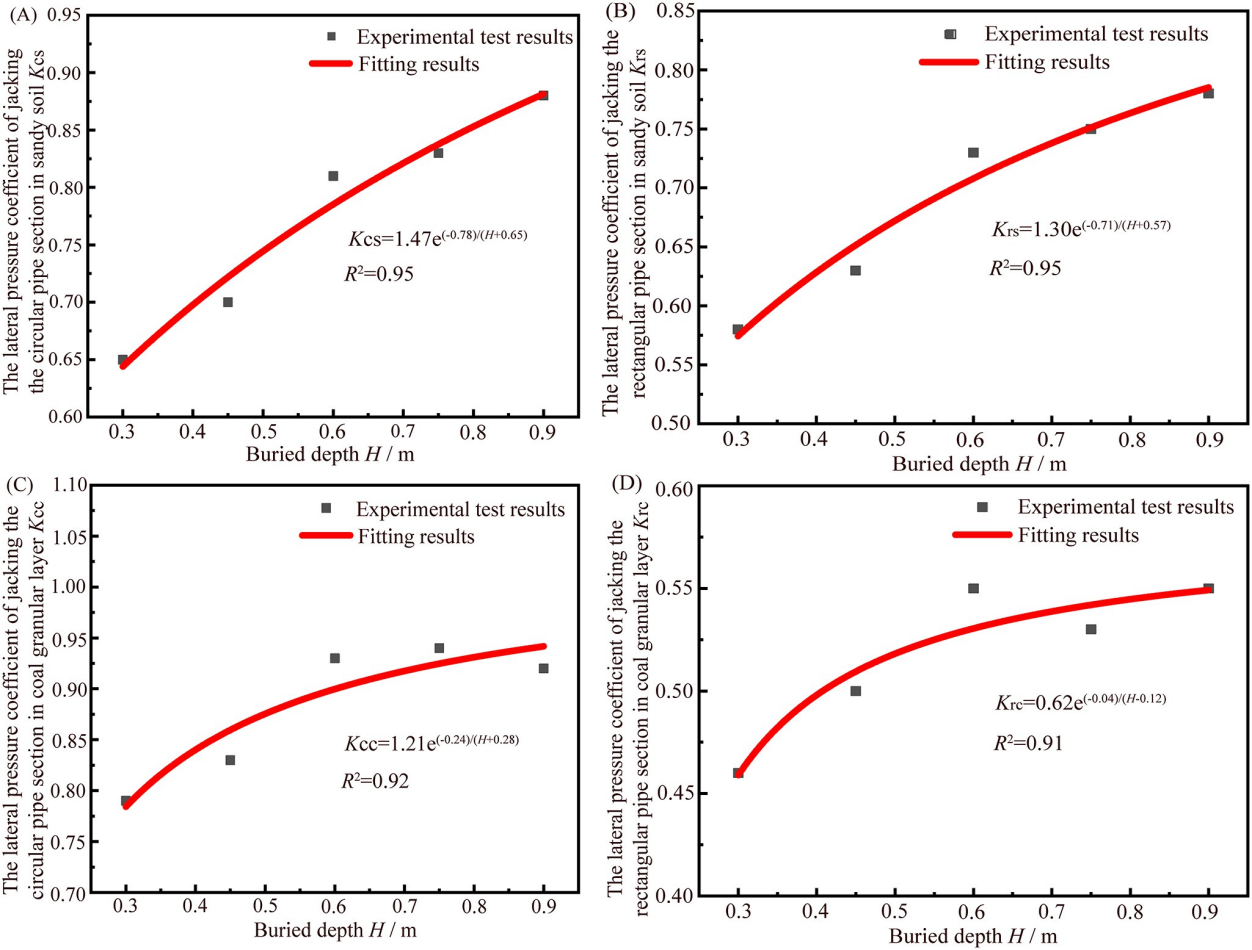
Fitting results of relationship between lateral pressure coefficient *K* and buried depth *H* of jacking circular and rectangular pipe sections in different simulated formations based on the results of experimental test and theoretical calculation equations. (A) The fitting results of relationship between lateral pressure coefficient *K* and buried depth *H* of jacking circular pipe section in sandy soil. (B) The fitting result of relationship between lateral pressure coefficient *K* and buried depth *H* of jacking rectangular pipe section in sandy soil. (C) The fitting result of relationship between lateral pressure coefficient *K* and buried depth *H* of jacking circular pipe section in coal granular layer. (D) The fitting result of relationship between lateral pressure coefficient *K* and buried depth *H* of jacking rectangular pipe section in coal granular layer.

The fitting results of relationship between lateral pressure coefficient *K* and buried depth *H* are shown in Fig 12. The variation rules of lateral pressure coefficient *K* and buried depth *H* under different experimental conditions are basically consistent, i.e., with the increase of buried depth *H*, the lateral pressure coefficient *K* shows a certain upward trend, and the upward trend is increasingly slow. The lateral pressure coefficient of jacking circular and rectangular pipe sections in sandy soil and coal granular layer are denoted as *K*_cs_, *K*_rs_, *K*_cc_ and *K*_rc_ respectively. The relationship equations between lateral pressure coefficient *K* and buried depth *H* under different experimental conditions are as follows. The corresponding goodness of fit are 0.95, 0.95, 0.92 and 0.91, so the results of data fitting are good.

(1) The lateral pressure coefficient of jacking the circular pipe section in sandy soil *K*_cs_:

$$K_{cs}=1.47e^{\left(-0.78)/(H+0.65\right)}$$ (8)

(2) The lateral pressure coefficient of jacking the rectangular pipe section in sandy soil *K*_rs_:

$$K_{rs}=1.3e^{\left(-0.71)/(H+0.57\right)}$$ (9)

(3) The lateral pressure coefficient of jacking the circular pipe section in coal granular layer *K*_cc_:

$$K_{cc}=1.21e^{\left(-0.24)/(H+0.28\right)}$$ (10)

(4) The lateral pressure coefficient of jacking the rectangular pipe section in coal granular layer *K*_rc_:

$$K_{rc}=0.62e^{\left(-0.04)/(H+0.12\right)}$$ (11)

Then, when Eqs (1) and (2) are used to calculate the pipe-soil frictional resistance, the lateral pressure coefficient *K* can be reasonably selected based on the pipe section shape and the formation type around the pipe section. Then, it is substituted into the theoretical calculation equations of pipe-soil frictional resistance, so as to modify the original Eqs (1) and (2). The modified theoretical calculation equations of pipe-soil frictional resistance under different conditions can be obtained as follows:

(1) The modified theoretical calculation equations of pipe-soil frictional resistance of jacking circular pipe section in sandy soil:

$$F_{cs}^{\prime }=fL[\frac{\pi }{2}\gamma D\left(1+K_{cs}\right)\left(H+\frac{D}{2}\right)-\frac{1}{3}\gamma D^{2}\left(2+K_{cs}\right)]+f\omega L$$ (12)

(2) The modified theoretical calculation equations of pipe-soil frictional resistance of jacking rectangular pipe section in sandy soil:

$$F_{rs}^{\prime }=fL\left[2\gamma Hl+2K_{rs}\gamma Hb+\gamma b^{2}K_{rs}\right]+f\omega L$$ (13)

(3) The modified theoretical calculation equations of pipe-soil frictional resistance of jacking circular pipe section in coal granular layer:

$$F_{cc}^{\prime }=fL[\frac{\pi }{2}\gamma D\left(1+K_{cc}\right)\left(H+\frac{D}{2}\right)-\frac{1}{3}\gamma D^{2}\left(2+K_{cc}\right)]+f\omega L$$ (14)

(4) The modified theoretical calculation equations of pipe-soil frictional resistance of jacking rectangular pipe section in coal granular layer:

$$F_{rc}^{\prime }=fL\left[2\gamma Hl+2K_{rc}\gamma Hb+\gamma b^{2}K_{rc}\right]+f\omega L$$ (15)

### Comparative analysis of pipe-soil frictional resistance based on the various research methods

To verify the accuracy of the above modified theoretical calculation equations, the pipe-soil frictional resistances of jacking circular and rectangular sections in sandy soil at different buried depth *H* are calculated by using them. The calculated results are compared with the results of the original theoretical calculation equations, the discrete element numerical simulation by EDEM software, experimental test, and the empirical estimation equations. Due to the limited space of this manuscript and the fact that many pipe jacking engineering practices are carried out in sandy soil, so only the conditions of jacking pipe sections in sandy soil are discussed.

### Comparative analysis on pipe-soil frictional resistance of jacking pipe sections based on the different theoretical calculation equations

The results of pipe-soil frictional resistance at different buried depth *H* can be calculated by the original and modified theoretical calculation equations are listed in Table 5. Moreover, the relative errors between the experimental test results (*F*_a_) and the various theoretical calculation results (*F*_c_, *F*_r_, *F′*_cs_ and *F′*_rs_) are also listed in Table 5.

**Table 5:** The pipe-soil frictional resistances calculated by different theoretical calculation equations.

Pipe section shape	Type of the theoretical calculation equations	Theoretical pipe-soil frictional resistance / N	Average pipe-soil frictional resistance by experimental test *F*_a_ / N	Relative error / %	Buried depth *H* / m
Circular	Original theoretical calculation Eq (1) *F*_c_	460	631	27.1	0.3
		673	930	27.63	0.45
		889	1304	31.83	0.6
		1103	1670	33.95	0.75
		1312	2006	34.6	0.9
	Modified theoretical calculation Eq (12) *F*′_cs_	630	631	0.16	0.3
		958	930	-3.01	0.45
		1311	1304	-0.54	0.6
		1669	1670	0.06	0.75
		2055	2006	-2.44	0.9
Rectangular	Original theoretical calculation Eq (1) *F*_r_	520	625	16.8	0.3
		770	927	16.94	0.45
		1020	1275	20	0.6
		1271	1603	20.71	0.75
		1521	1936	21.44	0.9
	Modified theoretical calculation Eq (12) *F*′_rs_	606	625	3.04	0.3
		949	927	-2.37	0.45
		1281	1275	-0.47	0.6
		1603	1603	0	0.75
		1991	1936	-2.84	0.9

### Comparative analysis on pipe-soil frictional resistance of jacking pipe sections based on EDEM software

**Fig 13:**
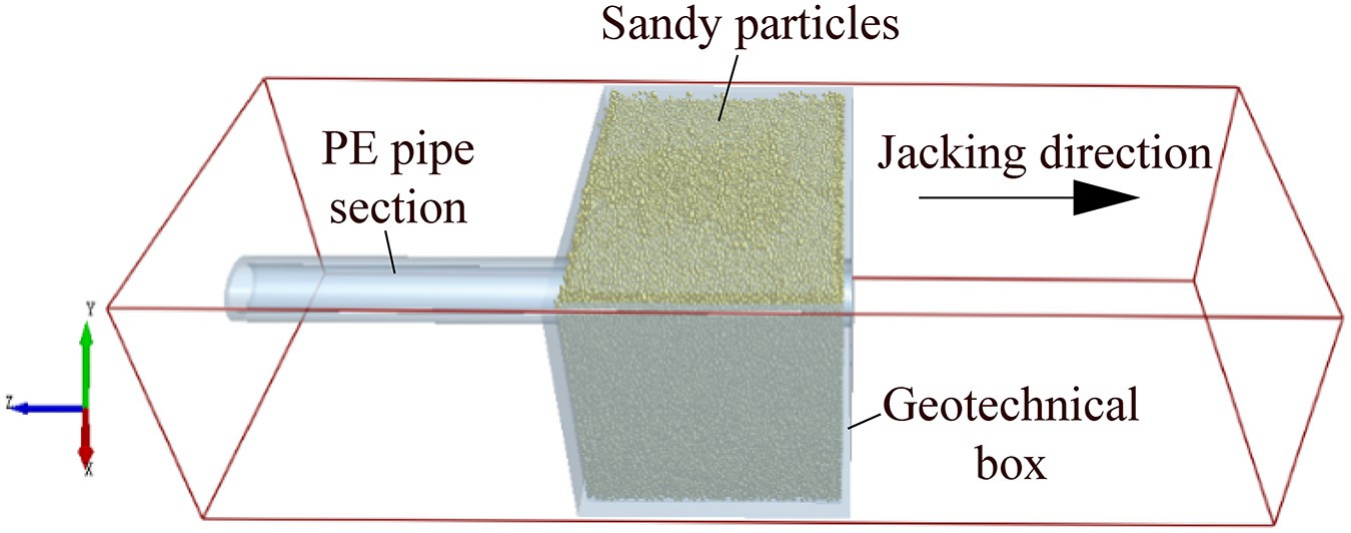
Numerical modeling of jacking pipe section in sandy soil built in EDEM software.

The discrete element method (DEM) was first proposed by Cundall and Strack, its core idea is to simulate the actual research object through many particle units, and to reflect the mechanical behavior of the solid structure or microstructure by solving the motion state of each particle. Currently, it has been widely used in geotechnical engineering, mining engineering, mechanical engineering, etc., especially for bulk material, such as soil particles [22–24, 26]. To further study the disturbance of pipe section to the soil around the pipe body in the process of pipe jacking, the discrete element numerical simulation analysis by EDEM software is carried out. The main analysis steps are as follows: ①The particle model and geometry (pipe sections and geotechnical box) modeling. The sandy soil is selected as the simulated formation, and the geotechnical box and pipe sections are modeled 1:1 based on the specifications of each part in the experimental test. The numerical modeling built in EDEM software as shown in Fig 13. ②Particle and geometry material creation and assignment. Because the main research focus is the frictional resistance generated by the contact between the pipe sections and soil, so the material model can be simplified. The physical parameters of pipe section and sandy particle mainly include intrinsic parameters such as Poisson’s ratio, shear modulus and density, as well as contact parameters such as collision recovery coefficient, static frictional coefficient and rolling frictional coefficient. Moreover, the contact model is used to numerically simulate the contact behavior between discrete bodies. Many different contact models are set up in EDEM software to simulate the contact between different kinds of materials. Among them, the contact model of Hertz-Mindlin (no slip) is developed on the basis of Hertz and Mindlin’s research to calculate the basic forces of particle-particle and particle-geometry contact. It is a basic contact model for numerical simulation of non-cohesive particles. Herein, the sandy particles are dry and have no obvious cohesion, and the wear effect of particles on the geometry is not considered. Thus, the contact model between particles and the basic model between particles and geometry can be adopted the contact model of Hertz-Mindlin (no slip). The detailed parameters for discrete element numerical simulation analysis in EDEM software can be obtained by referring to the references [25, 26] are listed in Table 6. ③The discrete soil particles generation. The particle generation is controlled to the experimental test height, and the energy-time curve is generated, so that the soil remains stable under the action of self-weight stress, i.e., the total energy is stable and unchanged. ④Set the jacking speed and jacking pipe sections at uniform speed. ⑤Post-processing: Generate the curves between the pipe-soil frictional resistance and the jacking distance.

**Table 6:** The detailed parameters for discrete element numerical simulation analysis in EDEM software [25, 26].

Parameters	Values
Density of pipe section / kg·m^-3^	950
Elastic modulus of pipe section / MPa	530
Poisson’s ratio of pipe section	0.4
Collision recovery coefficient between pipe section and sandy particles	0.31
Static frictional coefficient between pipe section and sandy particles	0.47
Rolling frictional coefficient between pipe section and sandy particles	0.3
Density of sandy particle	1861
Elastic modulus of sandy particle / Pa	20
Poisson’s ratio of sandy particle	0.22
Interparticle collision recovery coefficient	0.3
Interparticle static frictional coefficient	0.5
Interparticle rolling frictional coefficient	0.3

**Fig 14:**
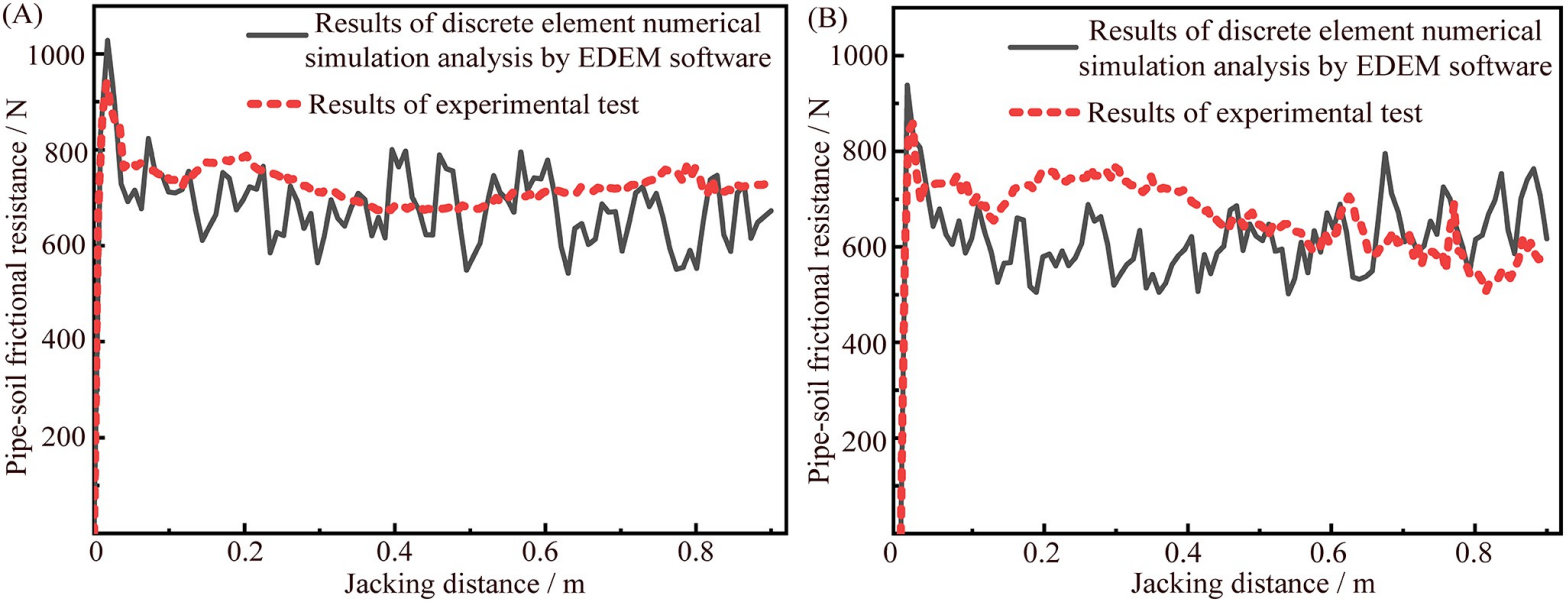
Relationship curves between the pipe-soil frictional resistance and the jacking distance of jacking circular and rectangular pipe sections based on the experimental test and the discrete element numerical simulation analysis by EDEM software at buried depth is 0.3 m. (A) Relationship curves between the pipe-soil frictional resistance and the jacking distance of jacking circular pipe section. (B) Relationship curves between the pipe-soil frictional resistance and the jacking distance of jacking rectangular pipe section.

The relationship curves between the pipe-soil frictional resistance and the jacking distance of jacking circular and rectangular pipe sections at buried depth *H* = 0.3 m based on the experimental test and the discrete element numerical simulation analysis by EDEM software as shown in Fig 14. The pipe-soil frictional resistance of the relationship curves obtained by the above two methods shows the same trend with the jacking distance, and the pipe-soil frictional resistance values have little difference, which indicates that it is feasible to carry out discrete element numerical simulation analysis on the experimental process of pipe jacking. Meanwhile, the accuracy of the results obtained by the experimental test is also indirectly demonstrated.

**Fig 15:**
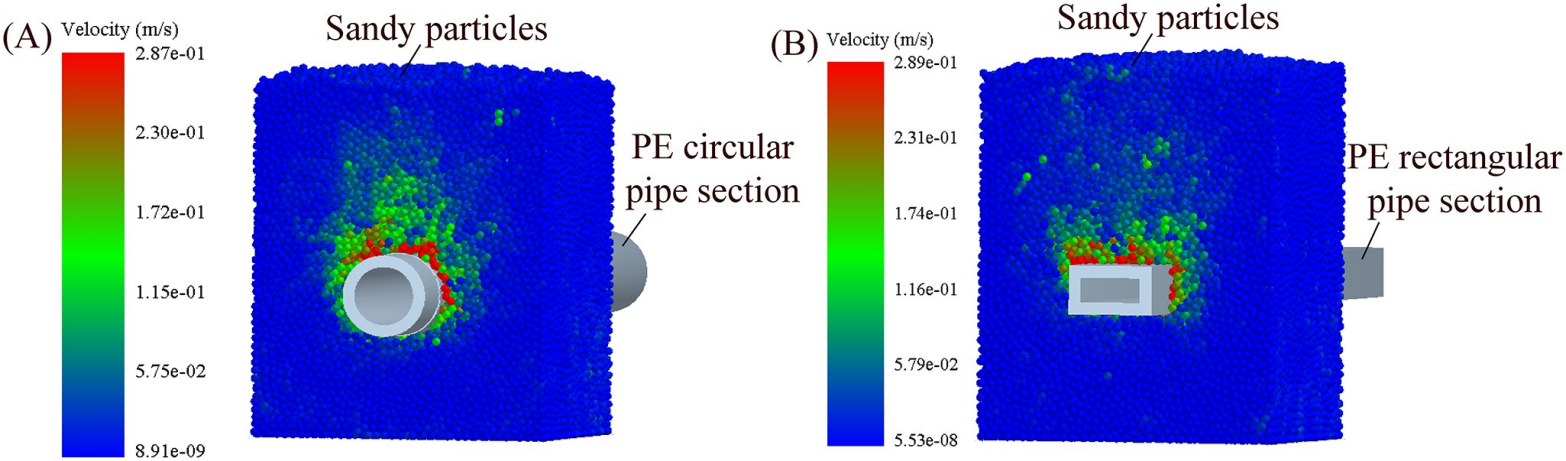
Disturbance of sandy particles around the pipe body during jacking different pipe sections at buried depth *H* = 0.3 m based on the discrete element numerical simulation analysis by EDEM software. (A) Disturbance of sandy particles around the pipe body during jacking circular pipe section. (B) Disturbance of sandy particles around the pipe body during jacking rectangular pipe section.

The disturbance of sandy particles around the pipe body during jacking circular and rectangular pipe sections at buried depth *H* = 0.3 m based on the discrete element numerical simulation analysis by EDEM software as shown in Fig 15. In the process of jacking pipe section, the disturbance range of the upper sandy particles is the largest, the disturbance range of the sandy particles on both sides of pipe section is the second, the disturbance range of the lower sandy particles is the smallest, and the sandy particles closer to the pipe section are disturbed more violently. Moreover, because of the influence on the shape of pipe section, the shapes of the disturbed area of sandy particles around pipe sections are different during jacking circular and rectangular pipe sections. The disturbance area of sandy particles above the pipe body is distributed in an arc shape during jacking circular pipe section, while the disturbance area of sandy particles above the pipe body is distributed in a concave shape during jacking rectangular pipe section. It is because of the different shape of pipe section that the disturbance around the pipe body is different in the process of pipe jacking, so as to the lateral pressure coefficient *K* used in calculating pipe-soil frictional resistance is also different.

The results of pipe-soil frictional resistance of jacking circular and rectangular pipe sections at different buried depth based on the discrete element numerical simulation analysis by EDEM software are listed in Table 7. Meanwhile, the relative errors between the experimental test results (*F*_a_) and the results of discrete element numerical simulation analysis by EDEM software (*F*_d_) are also listed in Table 7.

**Table 7:** The pipe-soil frictional resistances obtained by EDEM software.

Pipe section shape	Pipe-soil frictional resistance by EDEM software *F*_d_ / N	Average pipe-soil frictional resistance by experimental test *F*_a_ / N	Relative error / %	Buried depth *H* / m
Circular	688	631	-9.03	0.3
	882	930	5.16	0.45
	1111	1304	14.8	0.6
	1367	1670	18.13	0.75
	1641	2006	18.25	0.9
Rectangular	617	625	1.28	0.3
	845	927	8.85	0.45
	1065	1275	16.47	0.6
	1295	1603	19.21	0.75
	1525	1936	21.23	0.9

### Comparative analysis on pipe-soil frictional resistance based on the empirical estimation equations

Most of the existing empirical estimation equations of trenchless pipe jacking force and pipe-soil frictional resistance are aimed at circular pipe section, but there are few empirical estimation equations for rectangular pipe section [15–19, 27]. Herein, the focus is on the pipe-soil frictional resistance under full pipe-soil contact between pipe section and formation during trenchless pipe jacking. The head-on (penetration) resistance at the front end of the cutter head of pipe jacking machine is not considered, and the pipe-slurry-soil contact state when lubricating slurry is injected into the pipe-soil gap to form a slurry jacket is also not considered temporarily. Three empirical estimation equations of pipe-soil frictional resistance for jacking circular pipe section are summarized as follows:

(1) *Code for Construction and Acceptance of Water and Sewerage Pipeline Works* (GB50268-2008) proposes the following empirical estimation equation for pipe-soil frictional resistance [19]:

$$F_{e}=f\gamma D{\left[2H+(2H+D)K_{1}+\frac{\omega }{\gamma }D\right]}^{L}$$ (16)

(2) Qichang Xu proposes the following empirical estimation equation for pipe-soil frictional resistance [19]:

$$F_{e}=fK^{\prime }(HLD\;\;\;\gamma +\omega L)$$ (17)

(3) Huashen Tang proposes the following empirical estimation equation for pipe-soil frictional resistance [27]:

$$F_{e}=2\gamma Df\left[\left(\frac{\pi D}{8}+\frac{\pi b}{4f_{k}}\right)\left(1+K_{1}\right)+\frac{\pi D^{2}}{64bf_{k}}\left(3K_{1}-1\right)-\frac{D}{6}\left(K_{1}+2\right)\right]+\omega f$$ (18)

Where, *F*_e_ is the pipe-soil frictional resistance of jacking circular pipe section in the soil (N); *f* is the frictional coefficient between the outer wall of PE circular pipe section and the surrounding sandy soil, 0.474; *γ* is the volumetric weight of soil (N/m^3^); *D* is the outside diameter of circular pipe section (m); *H* is the thickness of covering soil above the top of pipe section (m); *L* is the length of contact between the outer wall of pipe section and soil (m); *K*_1_ is the coefficient of active earth pressure, *K*_1_ = tan^2^(45°-*φ*/2); *φ* is the internal frictional angle of sandy soil, 39.68°[28];*ω* is the linear density of pipe section (N/m); *K′* is the security coefficient, 1.5; *f*_k_ is the firmness factor of overlying sandy soil, 0.55; *b* is the width of earth pressure arch, *b* = *D*/2+*D*tan (45°-*φ*/2).

The results of pipe-soil frictional resistance calculated by the different empirical estimation equations are listed in Table 8. Meanwhile, the relative errors between the experimental test results (*F*_a_) and the results of different empirical estimation equations (*F*_e_) are also listed in Table 8.

**Table 8:** The pipe-soil frictional resistances obtained by the different empirical estimation equations.

Type of empirical estimation equations	Pipe-soil frictional resistance by empirical estimation equations *F*_e_ / N	Average pipe-soil frictional resistance by experimental test *F*_a_ / N	Relative error / %	Buried depth *H* / m
*Code for Construction and Acceptance of Water and Sewerage Pipeline Works* (GB50268-2008), Eq (16)	565	631	10.46	0.3
	841	930	9.68	0.45
	1108	1304	15.03	0.6
	1379	1670	17.43	0.75
	1650	2006	17.75	0.9
Qichang Xu, Eq (17)	351	631	44.37	0.3
	520	930	44.09	0.45
	685	1304	47.47	0.6
	851	1670	49.04	0.75
	1018	2006	49.25	0.9
Huashen Tang, Eq (18)	393	631	37.72	0.3
	393	930	57.74	0.45
	393	1304	69.86	0.6
	393	1670	76.47	0.75
	393	2006	80.41	0.9

## Discussion

**Fig 16:**
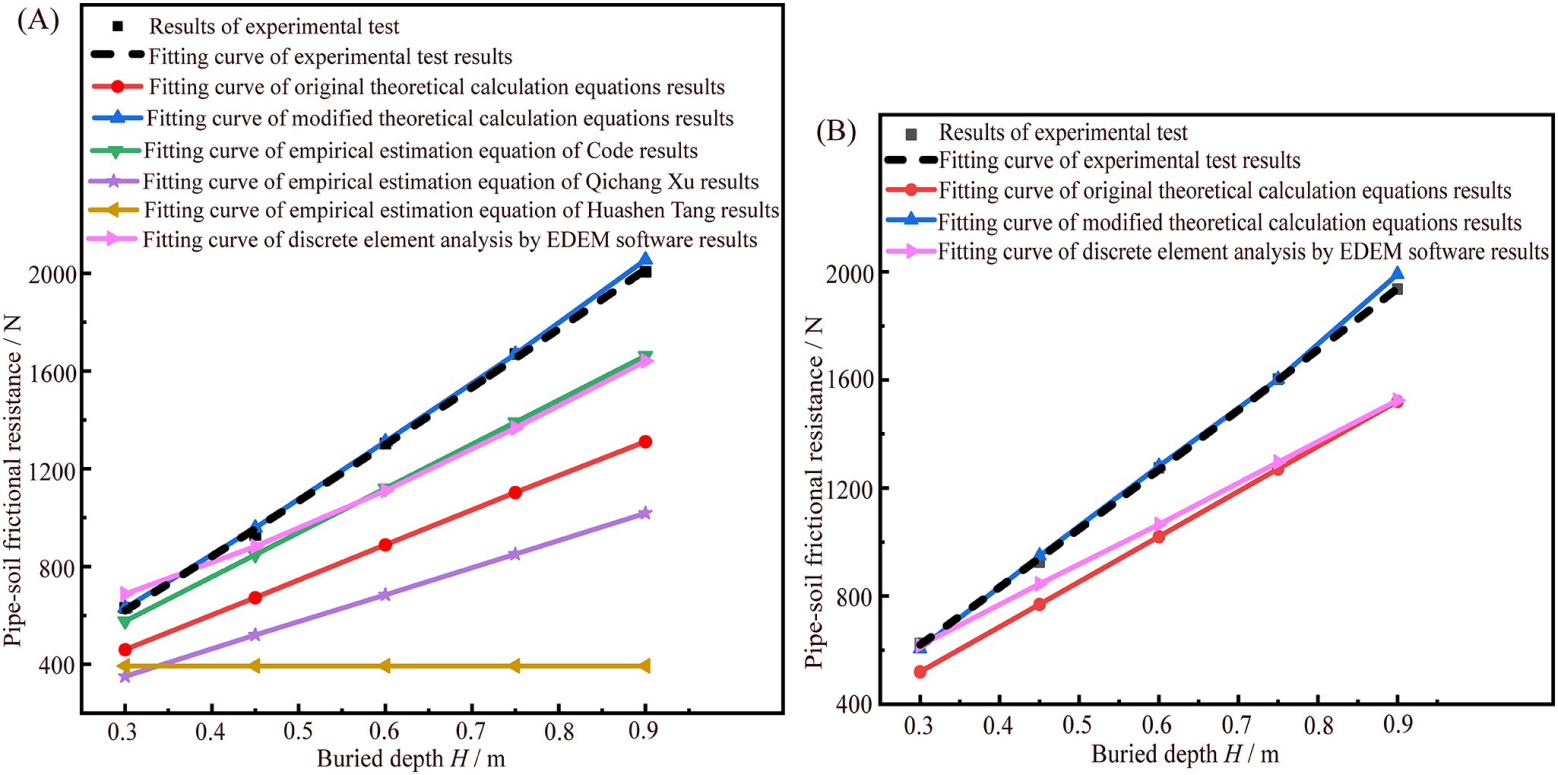
The relationship curves between the pipe-soil frictional resistance and the buried depth *H* based on the various research methods of jacking circular and rectangular pipe sections in sandy soil. (A) The relationship curves of jacking circular pipe section based on the various research methods. (B) The relationship curve of jacking rectangular pipe section based on the various research methods.

The relationship curves between the pipe-soil frictional resistance and the buried depth *H* based on the various research methods of jacking different pipe sections in sandy soil as shown in Fig 16. It can be seen from Fig 16(A) that the results of pipe-soil frictional resistance calculated by the empirical estimation equation of Huashen Tang is independent of buried depth *H*, because the pressure arch effect is considered in this equation, and it is believed that when there is a pressure arch in the soil above the pipe section, the earth pressure around the pipe section will not increase with the increase of the buried depth, i.e., the pipe-soil frictional resistance per unit length of pipe section is almost constant. Thus, the law of pipe-soil frictional resistance in this equation is different from that obtained by the experimental test, which also indirectly indicates that in the dry sandy soil, the earth pressure arch phenomenon is not easy to appear in the upper part of the pipe section. Moreover, the laws of pipe-soil frictional resistance obtained by the original and modified theoretical calculation equations, the empirical estimation equation of Qichang Xu, and the empirical estimation equation of code (GB50268-2008) are consistent with the law of experimental test results, i.e., the pipe-soil frictional resistance increases approximately linearly with the increase of pipe section buried depth *H*. The order of pipe-soil frictional resistance values obtained by the various research methods of jacking circular pipe section is as follows: the results of experimental test (*F*_a_) ≈ the results of modified theoretical calculation equation (*F′*_cs_) > the results of empirical estimation equation of *Code for Construction and Acceptance of Water Supply and Drainage Pipeline Engineering* (GB50268-2008) > the results of original theoretical calculation equation (*F*_c_) > the results of empirical estimation equation of Qichang Xu. Compared with the pipe-soil frictional resistance obtained by experimental test, the calculated values of the modified theoretical calculation equations have the smallest relative errors, while the calculated values of the original theoretical calculation equations have the large errors. The modified theoretical calculation equations can effectively improve the accuracy of the estimated pipe-soil frictional resistance. Moreover, the results obtained by the empirical estimation equation of *Code for Construction and Acceptance of Water Supply and Drainage Pipeline Engineering* (GB50268-2008) is the closest to the results of experimental test except for the modified theoretical calculation equations, but they are still smaller than the experimental test values. The reason is that the lateral pressure coefficient *K* used in the empirical estimation equation is also the coefficient of active earth pressure, which makes the calculation result small. In calculation, it can be modified with reference to the method in this paper. Furthermore, the results obtained by the empirical estimation equation of Qichang Xu are much smaller than the experimental test results, indicating that its security coefficient is relatively conservative, so it is often necessary to take a larger security coefficient to ensure that the estimated jacking force can make the smooth construction of pipe jacking.

It can be seen from Fig 16(B) that the order of pipe-soil frictional resistance values obtained by the various research methods of jacking rectangular pipe section is as follows: the results of experimental test (*F*_a_) ≈ the results of modified theoretical calculation equation (*F′*_rs_) > the results of original theoretical calculation equation (*F*_r_). Thus, the accuracy of the modified theoretical calculation equation is also illustrated.

As discussed above, no matter the pipe section shape is circular or rectangular, except for the results of pipe-soil frictional resistance calculated by the modified theoretical calculation equations, the results of pipe-soil frictional resistance obtained by the original theoretical calculation equations and the empirical estimation equations are smaller than the experimental test results, indicating that the original theoretical calculation equations and the empirical estimation equations may underestimate the actual pipe-soil frictional resistance in the practice of pipe jacking. It is very dangerous in the practice of pipe jacking, which may lead to the failure of pipe jacking, and even cause major engineering accidents. Relatively speaking, the modified theoretical calculation equations proposed in this paper can better estimate the pipe-soil frictional resistance. Thus, for the similar pipe jacking engineering, the modified theoretical calculation equations can be used to calculate the pipe-soil frictional resistance. However, for the other pipe jacking engineering, the relevant methods in this paper can be used for reference, and the corresponding parameters of the theoretical calculation equations can be reasonably modified to calculate, so as to ensure the smooth implementation of pipe jacking.

In conclusion, the pipe-soil frictional resistances of jacking circular and rectangular pipe sections in soil are different under the conditions without slurry. When calculating pipe-soil frictional resistance during pipe jacking construction, the relevant technical parameters and the correction coefficients should be reasonably selected based on the specific working conditions to ensure the smooth implementation of trenchless pipe jacking engineering practice.

## Conclusions

1. By comprehensive application of experimental test, theoretical calculation and discrete element numerical simulation analysis by EDEM software, the conditions of pipe-soil frictional resistance of circular and rectangular pipe sections during trenchless pipe jacking are compared and analyzed, and the several frequently used theoretical calculation equations and empirical estimation equations of pipe-soil frictional resistance for pipe jacking have been modified, which could provide some valuable references for the future scientific research, engineering practice, technological innovation and development of trenchless pipe jacking.
2. The independently developed multifunctional experimental apparatus for the pipe-soil frictional resistance testing, which is mainly composed of geotechnical box module, pipe section, jacking module and test module. It can be used to carry out the experimental research of pipe jacking under various complex formations and complex working conditions, which provides guidance for the jacking force design of pipe jacking engineering. According to the experimental tests of pipe-soil frictional resistance, the pipe-soil frictional resistances of jacking circular and rectangular pipe sections in coal granular layer are all smaller than in sandy soil. The relationships between the average pipe-soil frictional resistances *F*_a_ and the buried depths *H* of jacking circular and rectangular pipe sections in sandy soil and coal granular layer are obtained respectively, and the fitting relationship between lateral pressure coefficient *K* and buried depth *H* of jacking circular and rectangular pipe sections in sandy soil and coal granular layer are also obtained respectively, i.e., with the increase of pipe section buried depth *H*, the lateral pressure coefficient *K* shows an increasing trend, and the increasing range is smaller and smaller. Based on this, the modified theoretical calculation equations of jacking circular and rectangular pipe sections in sandy soil are presented.
3. Based on the discrete element numerical simulation analysis by EDEM software, the rationality of experimental test results can be verified, and the difference of soil disturbance around the pipe section affected by the carrying soil effect during the pipe jacking process can also be obtained, i.e., the disturbed area of the soil above the circular pipe section is distributed in an arc, while the disturbed area of the soil above the rectangular pipe section is slightly concave, which is also the reason why the value of the lateral pressure coefficient *K* in the original theoretical calculation equations of pipe-soil frictional resistance should be modified.
4. The differences of pipe-soil frictional resistance obtained by the results of experimental test, the results of modified theoretical calculation equations, the results of original theoretical calculation equations, and the results of empirical estimation equations are compared. There is little difference between the results of modified theoretical calculation equations and the experimental test, which shows the rationality of the modified theoretical calculation equations. However, the results of empirical estimation equations are relatively small, and some valuable suggestions are provided for the selection of calculation parameters in these equations.
5. In the practice of trenchless pipe jacking engineering, to reduce the pipe-soil frictional resistance, it is usually necessary to inject lubricating slurry (thixotropic mud) into the gap between the outer wall of pipe section and the formation to form slurry jacket. In the follow-up study, the experimental conditions after the slurry jacket are formed on the outer wall of pipe section by adding the lubricating slurry injection system will be further discussed, so as to make the physical model experiment more close to the actual pipe jacking engineering, so that the relevant research results can better serve the practice of trenchless pipe jacking engineering.
